# A Late Devonian Fertile Organ with Seed Plant Affinities from China

**DOI:** 10.1038/srep10736

**Published:** 2015-05-29

**Authors:** Deming Wang, Le Liu, Yun Guo, Jinzhuang Xue, Meicen Meng

**Affiliations:** 1 Key Laboratory of Orogenic Belts and Crustal Evolution, Department of Geology, Peking University, Beijing 100871, China; 2 Department of Geology, Yunnan University, Kunming 650091, Yunnan Province, China; 3 Institute of Geology, Chinese Academy of Geological Sciences, Beijing 100037, China

## Abstract

Seed plants underwent first major evolutionary radiation in the Late Devonian (Famennian), as evidenced by the numerous ovules described to date. However, the early pollen organs are underrepresented, so that their structure and evolution remain poorly known. Here we report a new taxon of pollen organ *Placotheca minuta* from the Late Devonian. The synangium consists of many basally and more or less laterally fused microsporangia borne on the margin of a pad. The prepollen is spherical and trilete. The appearance of Famennian synangia especially in *Placotheca* does not support the current understanding that the earliest pollen organs closely resembled the fructifications of the ancestral progymnosperms. *Placotheca* indicates earlier diversification of pollen organs than previously expected and is highly derived among the early pollen organs with trilete prepollen. It is suggested that, immediately after the origination of seed plants, pollen organs had evolved at a rapid rate, whereas their prepollen remained primitively spore-like.

On the basis of Early Carboniferous fossils, it was suggested that the earliest known pollen organs of seed plants (spermatophytes) may be morphologically very similar to the clusters of independent sporangia (their shape, size, dehiscence and wall) of Middle Devonian (Givetian)-Late Devonian (Frasnian) ancestral progymnosperms^1^,^2^. These similarities make it very difficult to estimate the diversification rate of the earliest pollen organs, which fundamentally relates to the fusion of sporangia and the formation of a synangial pad^2^. The earliest pollen organs occur in the Late Devonian (Famennian, 372-359 million years ago) but are extremely rare and typically too fragmentary to be identified^3^, when ovules or seeds are abundant and studied in detail and seed plants have undergone first major evolutionary radiation^3^,^4^,^5^,^6^,^7^. Although Famennian pollen organs have been described as synangiate^8^,^9^,^10^,^11^,^12^, they are simple because microsporangia in each organ are few and only basally fused and a pad is absent.

Now we present a new pollen organ, *Placotheca minuta* gen. et sp. nov., from the Late Devonian of Anhui Province, China. It has numerous basally and more or less laterally fused microsporangia on a bilaterally symmetrical pad. Thus, the synangium is unique and provides insight into the diversification of the earliest spermatophyte pollen organs, which are obviously different from the fructifications of the progymnosperms. *Placotheca* resembles the pollen organs of the lyginopterid spermatophytes, but it is more complex than most of the early pollen organs with trilete prepollen. In combination with Late Devonian and Carboniferous data, the new find suggests that the pollen organs and trilete prepollen of early seed plants had evolved at different rates.

## ? Division Spermatophyta

Class Lagenospermopsida

*Placotheca minuta* gen. et sp. nov.

### Etymology

The generic name from Greek plac and thēkē (meaning flat disc and container) refers to the shape of the pollen organ; the specific epithet from Greek miny (meaning small) denotes the size of the microsporangia.

### Holotype designated here

**Figure 1:**
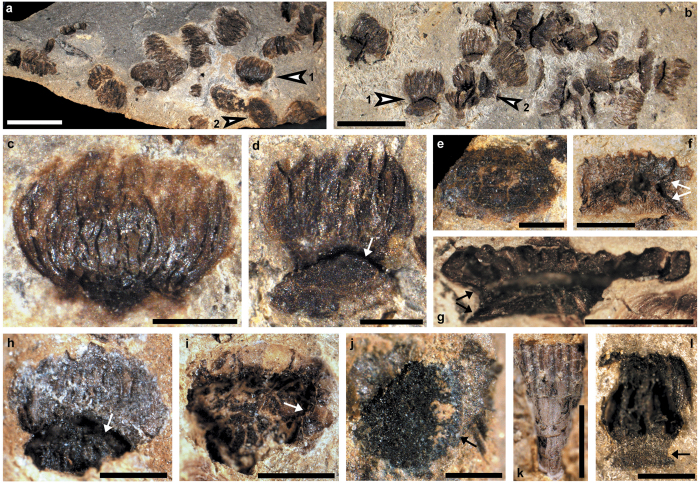
Pollen organs of *Placotheca minuta*. (**a**) Clusters of pollen organs, two synangia (arrows 1 and 2) enlarged in (**c**) and (**e**), respectively (PKUB13104). (**b**) Clusters of pollen organs, two synangia (arrows 1 and 2) enlarged in d and Supplementary Fig. 2d, respectively (PKUB13103). (**c**) An individual synangium with curved microsporangia. (**d**) Holotype, lateral view of synangium showing bottom of pad, gap (arrow) and microsporangia. (**e**) Face view of pad lacking margin. (**f,g**) Lateral view of synangium showing bottom and curved margin (arrows) of pad, and basal parts of microsporangia (PKUB13106, PKUB13104). (**h**) Lateral view of synangium showing pad, gap (arrow) and microsporangia (PKUB13101). (**i**) Face view of pad lacking microsporangia, showing gap (arrow) between bottom and curved margin of pad (PKUB13101). (**j**) Oblique face view of synangium showing curved margin (arrow) of pad and fragmentary microsporangia (PKUB13106). (**k**) More or less laterally fused microsporangia arising from margin of pad (PKUB13101). (**l**) Curved microsporangia arising from margin (arrow) of pad (PKUB13108). Scale bars, (**a**,**b**) 2 mm, (**c-f**,**h**,**j**,**l**) 0.5 mm, (**g**,**i**,**k**) 1 mm.

PKUB13103 (Department of Geology, Peking University, Beijing), a pollen organ with microsporangia and synangial pad (Fig. 1d).

### Paratypes

PKUB13104, a synangial pad (Fig. 1e), and a synangium with margin of synangial pad bearing microsporangia (Fig. 1g); PKUB13101, a synangium with margin of pad (Fig. 1i); PKUB13101, microsporangia connected to margin of pad (Fig. 1k); PKUB13108, microsporangia attached to margin of pad (Fig. 1l).

### Locality and horizon

Xiangkou, Dongzhi County, Anhui Province, China; Upper Devonian (Famennian) Wutong Formation.

### Diagnosis

Synangiate pollen organ bilaterally symmetrical; oval synangial pad with dome-shaped bottom, with margin curving toward and then extending away from synangial center. Microsporangia numerous, elongate, arising from margin of pad to produce a single ring, with their tips curving toward synangial center; microsporangia basally and somewhat laterally fused and distally free. Prepollen trilete, small, spherical in shape, and bearing indistinct papillate ornamentation.

The fossils come from the Wutong Formation at the Xiangkou section, Xiangyu Town, Dongzhi County, Anhui Province, South China. This formation, widespread in the lower reaches of Yangtze River including Anhui, is subdivided into the lower Guanshan Member, consisting mainly of quartzose sandstone and conglomerate, and the upper Leigutai Member, characterized by interbedded quartzose sandstone and mudstone^13^. Assemblages of plants, spores, fish and conchostracans indicate that the Wutong Formation is Upper Devonian (Famennian), and that the Guanshan Member is lower Famennian, the Leigutai Member upper Famennian^13^,^14^. The progymnosperm *Archaeopteris halliana* occurs in the basal part of the Leigutai Member^15^ and *Placotheca* is found ca. 11 m above this horizon (Supplementary Fig. 1). From a thin layer of silty mudstone, we obtained eight specimens containing about 200 pollen organs preserved as impressions and compressions.

Synangiate pollen organs occur in aggregates (Fig. 1a,b) and are 1.6-2.2 mm long, 0.8-1.9 mm wide and 1.5-1.8 mm high. Individuals are often incompletely preserved but show many microsporangia (Fig. 1c and Supplementary Fig. 2a). The base of the synangium forms an oval pad or cushion (Fig. 1e and Supplementary Fig. 2b), which is 1.2-1.4 mm long and 0.6-0.9 mm wide and represents bilateral symmetry. The bottom of the pad is dome-shaped and 0.3-0.4 mm high (Fig. 1d,f,g, Supplementary Fig. 2c), with microsporangia arranged in a single row around the circumference of the pad (Fig. 1d,f-h, Supplementary Fig. 2d and 3a). The margin of the pad first curves toward (Fig. 1f, lower arrow, g, lower arrow, Supplementary Fig. 3b,c) and then extends away from the synangial center (Fig. 1f, upper arrow, g, upper arrow) to join the bases of the microsporangia. The pad margin is 0.3-0.5 mm high (Fig. 1f,g,k,l, arrow, Supplementary Fig. 2f) and up to 6.3 mm in circumference (Fig. 1i). The reverse side of the pad with curved margin (Fig. 1j, arrow) is visible. A gap is frequently observed between the bottom and curved margin of the pad (Fig. 1d, arrow, f,g,h, arrow, i, arrow, Supplementary Fig. 2d, arrow, e, arrow, Supplementary Fig. 3a–c).

**Figure 2:**
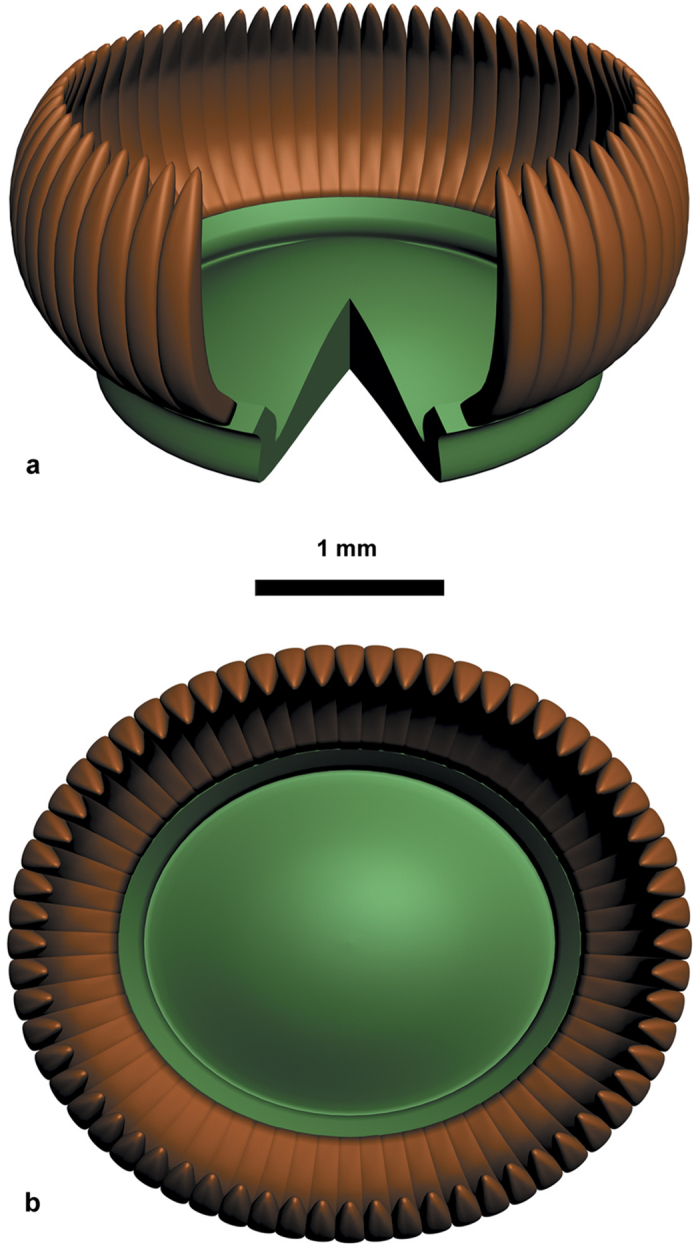
Reconstruction of pollen organ of *Placotheca minuta*. **(a)** Lateral view of synangium, with part of pad and several microsporangia removed to show details. **(b)** Face view of synangium.

Arising from the margin of the pad (Fig. 1f,g,k,l, Supplementary Fig. 2d and 3d), the microsporangia extend away from and then curve toward the synangial center (Fig. 1c,g,l, Supplementary Fig. 2a). Microsporangia are basally and more or less laterally fused (Figs. 1c,d,k,l, Supplementary Fig. 2a,d), distally free (Fig. 1c,d) and in a single ring (Fig. 1d,h,k). Microsporangia are elongate, 0.9-1.3 mm long and 0.09-0.12 mm wide. Although preservation generally precludes a direct counting of all of the microsporangia in a synangium, on the basis of the circumference of the pad margin and the average width of a microsporangium, a single pollen organ is estimated to possess about 60 microsporangia (Fig. 2a,b).

**Figure 3:**
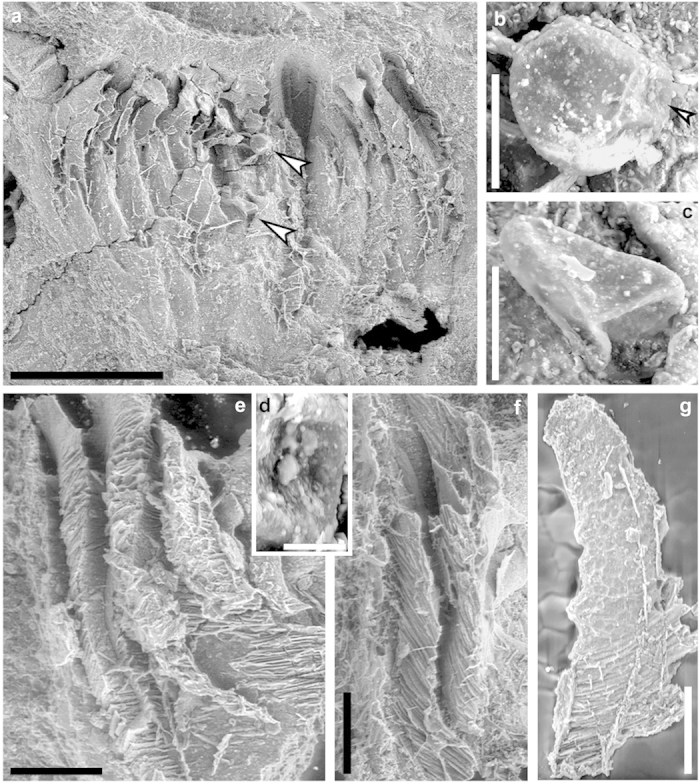
SEM (scanning electronic microscopy) observation of microsporangia and prepollen of *Placotheca minuta*. (**a**) Elongate microsporangia and two prepollen (upper and lower arrows) enlarged in (**b**) and (**c**), respectively (PKUB13104). (**b,c**) Spherical or triangular prepollen in proximal view and with trilete rays, arrow showing part enlarged in (**d**). (**d**) Prepollen exine bearing papillate ornamentation. (**e**) Three more or less laterally fused microsporangia with wall containing parallel ridges. (**f**) Microsporangium wall containing parallel ridges. (**g**) Microsporangium wall with parallel ridges and isometric cells. Scale bars, (**a**) 0.5 mm, (**b,c**) 50 μm, (**d**) 10 μm, (**e-g**) 0.1 mm.

The microsporangial wall contains parallel ridges oriented horizontally or obliquely (Fig. 3e-g). From inside view of the microsporangium, the wall layer consists of isometric cells with diameter of 7.0-13.0 μm (Fig. 3g). Dehiscence of the microsporangia is unclear. Only two prepollen grains have been observed in a pollen organ (Fig. 3a, arrows) and they probably belong to the adjacent microsporangia. Prepollen is ca. 60 μm in diameter and spherical (Fig. 3b), although a triangular shape may result from preservation (Fig. 3c). The trilete rays are 44-50 μm long. The exine is ornamented with indistinct papillae of 1.0-2.0 μm diameter (Fig. 3d).

The earliest known spermatophyte pollen organs are Late Devonian (Famennian) *Elkinsia polymorpha*^8^, *Telangium schweitzeri*^9^, *Telangiopsis* sp.^10^ and *Cosmosperma polyloba*^12^, which have been found in association with ovules. They have been described with synangium comprising up to eight microsporangia fused only at the base. *Kongshania synangioides* has synangium consisting of six basally fused microsporangia^11^. The microsporangia of these Famennian synangia are 1.6-8.0 mm long and 0.14-1.80 mm wide, comparatively larger than those of *Placotheca*.

It has been noted that some ferns (marattialeans) may possess synangia with trilete grains which superficially resemble synangia of the lyginopterid spermatophytes, but they have never been known prior to the Carboniferous and the synangia are foliar-borne^4^,^16^,^17^. In contrast, the synangia of *Placotheca* are from Late Devonian, occur with some ovules in the same bed of sediments, and have not been found in association or attachment with any laminate pinnules. The pad of these synangia is oval and characterized by a dome-shaped bottom and a curved margin. The pad with such a shape and structure differs clearly from a pinnule with the abaxial surface attached by synangia or sori as in marattialeans. Sometimes, the synangia of marattialeans are partially enclosed by the pinnules^18^. It is thus unlikely that so many (hundreds of) detached synangia in our study have separated from the pinnules if *Placotheca* has such leafy organs. The tip of the dome may represent the point of insertion of a stalk on the lower surface of the pad. The Famennian pollen organs are borne distally at stalks or often found detached^8^,^9^,^10^,^11^,^12^. Consequently, we prefer to interpret the synangia of *Placotheca* as pollen organs of seed plants. The stalks unfortunately have not been preserved with the pollen organs and few in situ prepollen have remained, suggesting that the synangia had abscised from parent plant after shedding most prepollen.

Many Early Carboniferous fructifications with trilete prepollen (e.g., *Zimmermannitheca*, *Geminitheca* and *Simplotheca*) possess clusters of terminal, elongate and independent sporangia. Prior to the discovery in Late Devonian, it was proposed that the earliest spermatophyte pollen organs were nonsynangiate and morphologically indistinguishable from the fructifications of the ancestral aneurophyte progymnosperm^1^,^2^. Nevertheless, currently available Famennian pollen organs, especially *Placotheca*, are synangiate and clearly distinct from progymnosperm fructifications.

Late Devonian pollen organs are few and simple, whereas the contemporaneous ovules are numerous and demonstrate morphological diversification in many kinds of cupules and integuments^3^,^4^,^12^. The large number of small and somewhat laterally fused microsporangia born on a synangial pad makes the structure of *Placotheca* unusual among the earliest pollen organs during the Famennian. *Placotheca* indicates earlier diversification of pollen organs than previously thought.

Lyginopterid spermatophytes were widespread in the Carboniferous^4^. Their pollen organs are characterized by radially or bilaterally symmetrical synangia, usually with few and partially fused microsporangia (1.5-5.5 mm long and 0.2-1.5 mm wide) often surrounding a central cavity; prepollen is spherical and trilete, normally less than 70 μm in diameter and bears inconspicuous ornamentation on the sexine^1^,^2^,^9^. On the basis of the number, arrangement and fusion of microsporangia, the Carboniferous synangiate pollen organs with trilete prepollen may be classified as simple, aggregate and compound types^2^. *Placotheca* conforms to most features of lyginopterid spermatophyte pollen organs, but each synangium has numerous more extensively fused microsporangia than lyginopterid synangia of the simple type (e.g., *Telangium*, *Telangiopsis* and *Feraxotheca*) or aggregate type (e.g., *Phacelotheca* and *Schopfiangium*). Individual synangia of *Placotheca* are comparable in microsporangial number and fusion to the compound type (*Crossotheca*). Therefore, if represents pollen organs of the seed plants, *Placotheca* is more complex than all Late Devonian, and even many Carboniferous synangia with trilete prepollen.

Small trilete prepollen characterizes Famennian spermatophytes (e.g., *Elkinsia polymorpha*^8^, *Telangium schweitzeri*^9^ and *Placotheca*) and many Carboniferous ones^1^,^2^,^4^,^19^. The uniform prepollen is primitive because of resembling progymnosperm spores in spherical shape, trilete rays and undeveloped ornamentation^2^. The prepollen is only functionally different from such spores^4^. Compared to the prepollen at an extremely low evolutionary rate, the pollen organs quickly became highly derived and diversified after the spermatophytes had originated from the progymnosperms.

## Methods

### Material

All specimens have been deposited in Department of Geology, Peking University, Beijing, China.

### Preparing and imaging

Steel needles were used to expose some pollen organs, whose photographs were made with a digital camera and microscope. Several pollen organs, a number of microsporangia and a few prepollen were observed with scanning electronic microscopy.

## Additional Information

**How to cite this article**: Wang, D. M. *et al*. A Late Devonian fertile organ with seed plant affinities from China. *Sci. Rep*. **5**, 10736; doi: 10.1038/srep10736 (2015).

## Supplementary Material

Supplementary Information
