# Supplementary material for: A Late Devonian Fertile Organ with Seed Plant Affinities from China
Source: Sci Rep. 2015 May 29;5:10736. doi: 10.1038/srep10736 (PMC4448223; doi:10.1038/srep10736)
Supplement: Supplementary Information [file srep10736-s1.pdf]

A Late Devonian fertile organ with seed plant affinities from China

Deming Wang[1*], Le Liu[1], Yun Guo[2], Jinzhuang Xue[1*] & Meicen Meng[3]

[1]Key Laboratory of Orogenic Belts and Crustal Evolution, Department of Geology, Peking University, Beijing 100871, China.

[2]Department of Geology, Yunnan University, Kunming 650091, Yunnan Province, China.

[3]Institute of Geology, Chinese Academy of Geological Sciences, Beijing 100037, China.

[*]e-mail: dmwang@pku.edu.cn; pkuxue@pku.edu.cn

Supplementary Figure 1 Stratigraphic column at the Xiangkou section, Dongzhi County (Anhui, China). The positions of the Upper Devonian (Famennian) plants including *Placotheca minuta* gen. et sp. nov. Fm.=Formation.

Supplementary Figure 2 Pollen organs of *Placotheca minuta*. (**a**) Curved microsporangia (PKUB13104). (**b**) Face view of oval pad (PKUB13101). (**c**) Lateral view of dome-shaped bottom of pad (PKUB13104). (**d**) Oblique face view of synangium, showing pad, gap (arrow) and incomplete microsporangia (PKUB13103). (**e**, **f**) Synangium before and after dégagement, showing gap (arrow), microsporangia and exposed margin (PKUB13107). Scale bars, (**a, b, d**) 0.5 mm, (**c**) 0.2 mm, (**e, f**) 1 mm.

Supplementary Figure 3 Pollen organs of *Placotheca minuta*. (**a-d**) Line drawings of synangium in Fig. 1d, f, g, l, respectively. Horizontal lines: boundary between two adjacent microsporangia; oblique lines: gap between margin and bottom of pad; dashed line: position where synangium is fractured or hidden by rock matrix.

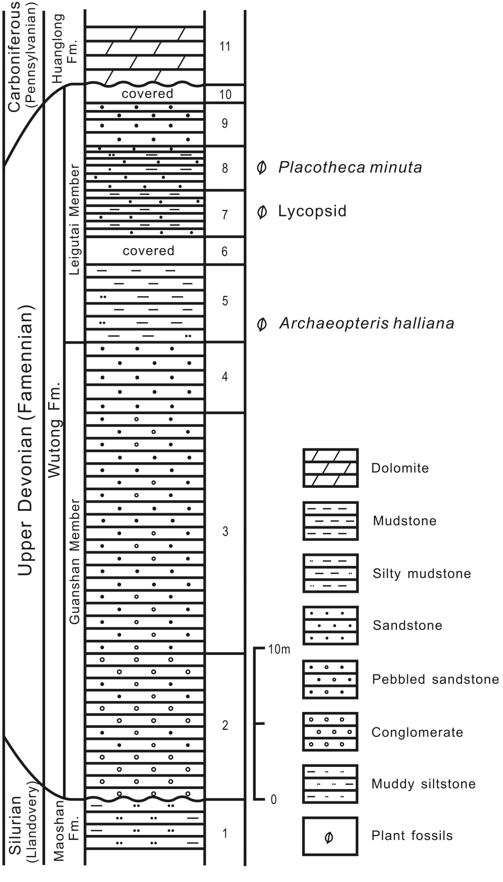

Carboniferous (Pennsylvanian)
Upper Devonian (Famennian)
Silurian (Llandovery)
Huanglong Fm.
Wutong Fm.
Maoshan Fm.
Leigutai Member
Guanshan Member
covered
covered
11
10
9
8
7
6
5
4
3
2
1
Placotheca minuta
Lycopsid
Archaeopteris halliana
10m
0
Dolomite
Mudstone
Silty mudstone
Sandstone
Pebbled sandstone
Conglomerate
Muddy siltstone
Plant fossils

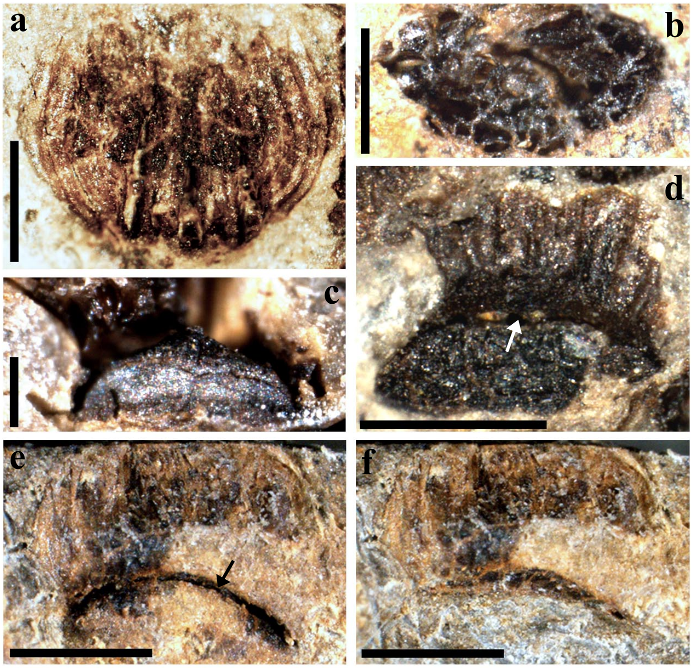
a
b
c
d
e
f

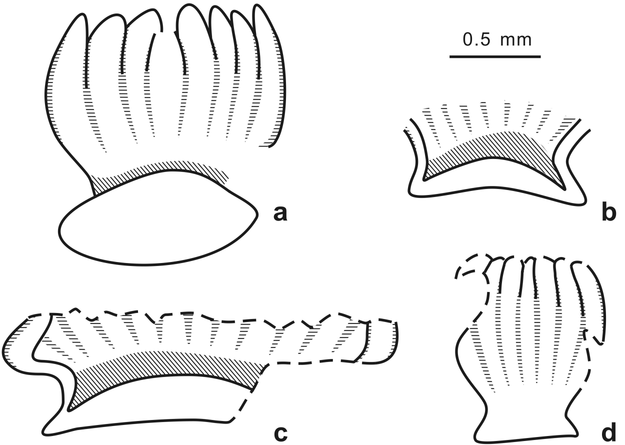
0.5 mm
a
b
c
d
